# Multimodal deep representation learning for protein interaction identification and protein family classification

**DOI:** 10.1186/s12859-019-3084-y

**Published:** 2019-12-02

**Authors:** Da Zhang, Mansur Kabuka

**Affiliations:** Department of Electrical and Computer Engineering, University of Miami, Coral Gables, FL U.S.

**Keywords:** Protein-protein interaction network, Multimodal deep neural network, Knowledge graph representation learning

## Abstract

**Background:**

Protein-protein interactions(PPIs) engage in dynamic pathological and biological procedures constantly in our life. Thus, it is crucial to comprehend the PPIs thoroughly such that we are able to illuminate the disease occurrence, achieve the optimal drug-target therapeutic effect and describe the protein complex structures. However, compared to the protein sequences obtainable from various species and organisms, the number of revealed protein-protein interactions is relatively limited. To address this dilemma, lots of research endeavor have investigated in it to facilitate the discovery of novel PPIs. Among these methods, PPI prediction techniques that merely rely on protein sequence data are more widespread than other methods which require extensive biological domain knowledge.

**Results:**

In this paper, we propose a multi-modal deep representation learning structure by incorporating protein physicochemical features with the graph topological features from the PPI networks. Specifically, our method not only bears in mind the protein sequence information but also discerns the topological representations for each protein node in the PPI networks. In our paper, we construct a stacked auto-encoder architecture together with a continuous bag-of-words (CBOW) model based on generated metapaths to study the PPI predictions. Following by that, we utilize the supervised deep neural networks to identify the PPIs and classify the protein families. The PPI prediction accuracy for eight species ranged from 96.76% to 99.77%, which signifies that our multi-modal deep representation learning framework achieves superior performance compared to other computational methods.

**Conclusion:**

To the best of our knowledge, this is the first multi-modal deep representation learning framework for examining the PPI networks.

## Backgrounds

Protein-protein interaction (PPI) networks are becoming increasingly crucial for analyzing biomedical functions, retrospecting species evolution and analyzing different compounds that cause diseases. Moreover, comprehending the intrinsic patterns behind PPI networks facilitates the understanding of cancer-related protein-protein interfaces and the topological structures of the cancer networks. Normally, two groups of research methods can be formulated when analyzing PPI networks: computational biology methods and high-throughput experimental methods. Given a PPI network, computational biology methods calculate the distances between proteins according to network theory metrics (e.g. betweenness, centrality, average degree) or machine learning algorithms[1–3]. High-throughput techniques, on the contrary, including yeast two-hybrid screens (Y2Hs)[4], mass spectrometry protein complex identification (MS-PCI) [5] and Nuclear Magnetic Resonance (NMR)[6], etc. pro- duce large amounts of data for constructing primary protein databases. These databases provide primary and rich sources for developing molecular and functional networks. Nevertheless, these genome-based techniques demand expensive wet-lab investment and exhaustive lab work. Also, because of the equipment biases in the experimental environment, the results generated by these genome-based methods are subjected to inevitable inaccuracy. Moreover, compared with the significant amount of protein sequence data, the functional units that have been discovered are comparatively restricted. Previously, traditional machine learning algorithms such as decision trees (DT), naive bayes (NB) and nearest neighbor (NN)[7] have been utilized efficiently in lots of data mining tasks. Yet, these traditional machine learning techniques lack the capacity of discovering hidden associations and extracting discriminant features from the input complex data. Lately, accompanied with the advancement of AI techniques, deep learning methodologies[8] extracting non-linear and high dimensional features from the protein sequences [9, 10] have emerged as a new tendency. These deep learning techniques and frameworks have been recently applied in tremendous biomedical research fields, biological network analysis, and medical image examination. However, since natural and real-world data distributions are highly complex and multimodal, it is essential to incorporate different modalities and patterns from the data to attain satisfactory performance. Additionally, discovering biological pattern from the graph topology of these protein networks is fundamental in comprehending the functions of the cells and their constitutional proteins. When applying deep learning techniques to biological network analysis, these modalities include topological similarities such as 1*st*-order similarity, 2*nd*-order similarity, and homology features extracted from protein sequences. Additionally, next-generation sequencing technologies also generate large amounts of DNA/RNA sequences which are then translated into protein peptides in the form of stacked amino acid residues. These protein sequences consist of fundamental molecules which perform biological functions for various species [11–13]. Thus, the functionality of a protein is encoded in the amino acid residues. To recognize the protein functionalities, researchers categorize proteins into various families such that proteins within the same family share similar functions or become the parts on the same pathway. In this paper, we propose a advanced multi-modal deep representation learning framework preserving different modalities to harvest both protein sequence similarity and topological proximity. This framework leverages both relational and physicochemical information from proteins and successfully integrates them using a late feature fusion technique. These concatenated features are provided to the interaction identifier and protein family classifier for the training and testing tasks.

To the best of our knowledge, this is the first multi-modal deep representation learning framework for analyzing protein-protein interaction networks. Specifically, the contributions of our method are listed as follows:

- A novel multi-modal deep representation learning framework is presented that integrates both unsupervised learning and supervised learning to predict Protein-protein interactions and identify protein families.
- In the unsupervised learning phase, we integrate the multi-modality features learned from Continuous Bag of Word (CBOW) model based on generated metapaths and a stacked auto-encoder (SAE) model to combine topological proximity features and the physicochemical sequence features for each protein. The SAE model is effective when denoising the systems and is capable of reconstructing useful representations from the partial raw data.
- In the supervised learning phase, we feed the output from the unsupervised model into the supervised model and achieve the higher PPI prediction accuracy and protein family classification accuracy. We apply our model on the DIP and the HPRD networks to formulate low-dimensional representations for high-level protein features.

The remainder of the paper is organized as follows. We present the data preprocessing strategies, theoretical background and algorithms of our methods in the “Methods” section. The “Results” section describes the framework parameter settings, dataset statistics, and experimental results. Finally, we conclude the paper and envision the future work in the conclusion part.

## Methods

In this section, we illustrate our proposed framework which can be divided into three phases including a protein sequence preprocessing phase, an unsupervised learning phase, and a supervised learning phase. Comprehensive illustrations of each phase associated with their inputs and outputs are examined in the following sections.

### Protein sequence preprocessing phase

For computational intelligent machine learning and data mining methods, it is demanded that the lengths of the feature dimensions are the same. Consequently, encoding protein sequences with various length amino acids into equivalent length feature vectors are necessary for the following machine learning tasks. Therefore, in this phase, we extract physicochemical information from the protein residues consisting of stacked amino acids and transform them into equal length numerical vectors. In this procedure, we maintain the constitutional protein residue information as much as possible by obtaining the inherent information in the protein peptides. We use the following four methods for converting various lengths protein sequences into fixed length numerical vectors[14].

#### Amino Acid Composition

The amino acid composition(AAC) statistics is the proportion of each amino acid type inside a protein sequence. The AAC computes the ratio of each type of amino acid and convert the peptides into equal length numerical vectors. The AAC can be computed as follows:

1 $$ fr(t) = \frac{N(t)}{N}, t\in \{A,C,D,\dots Y\}  $$

Here, *N*(*t*) is the number of amino acid type *t* in a protein sequence with length *N* and {*A*,*C*,*D*,…*Y*} represents twenty types of amino acids.

#### Grouped Amino Acid Composition(GAAC)

For the Grouped Amino Acid Composition, the 20 types of amino acids are classified into five categories according to their physicochemical properties[15]. These five categories include the aliphatic group (g1: GAVLMI), aromatic group (g2: FYW), positive charge group (g3: KRH), negative charged group (g4: DE) and uncharged group (g5: STCPNQ)[14]. GAAC computes the frequency of each group of amino acids as follows:

2 $$\begin{array}{@{}rcl@{}} f(g) = \frac{N_{g}}{N}, g\in \{g1, g2,g3,g4,g5\} \end{array} $$

3 $$\begin{array}{@{}rcl@{}} N(g_{t}) = \sum{N(t)}, t\in \{g\} \end{array} $$

Here, *N*_*g*_ is the number of amino acids in group *g*, *N*(*t*) is the number of amino acid for type *t*, and *N* is the total length of the peptide sequence.

#### Conjoint Triad

The Conjoint Triad(CT) takes into account the properties of one amino acid and its adjacent amino acids by considering three adjoining amino acids as an individual feature[16]. We first represent the protein sequence using a binary space (*V*,*F*). For the amino acids that have been categorized into 7 classes[16], the length of *V* can be computed as 7×7×7=343. Therefore, the dimension of vector *V* is 343. Each cell *V*_*i*_ ∈*V* indicates a triad feature. *F* is the number of vectors corresponding to *V*. *f*_*i*_ is the value of the *ith* dimension of *F* representing the number of types *V*_*i*_ appearing in the protein sequence. Therefore, the *CT* descriptor for a protein sequence can be derived as follows:

4 $$ d_{i} = \frac{f_{i}-min\{f_{1},f_{2},\dots, f_{343}\}} {max\{ f_{1}, f_{2},\dots f_{343} \}}  $$

Here, *d*_*i*_ is the normalization of *f*_*i*_.

#### Quasi-Sequence-Order

For each amino acid type, a quasi-sequence-order descriptor can be defined as the following equation:

5 $$ X_{r} = \frac{f_{r}}{\sum_{1}^{20}f_{r}+w\sum_{d=1}^{nlag}\tau_{d}}, r=1,2,\dots, 20  $$

Here, *f*_*r*_ is the normalized occurrence of amino acid type *r* and *w* is the weighting factor initialized at *w*=0.1[14]. *nlag* is the maximal value of the looking back parameter *lag*. $\tau _{d}=\sum _{i=1}^{N-d}(d_{i,i+d})^{2}$, *d*=1,2,3,…,*nlag*, and *d*_*i*,*i*+*d*_ is the distance between cell *d*_*i*_ and *d*_*i*+*d*_ given a distance matrix. In the experiments, we set the default value of the *lag* at 30.

### Unsupervised Learning Phase

After preprocessing the raw sequential data, we transform various lengths of protein sequences into 468 equal length vectors using iFeature APIs [14]. In the unsupervised learning phase, we first extract the deep features from previously generated equal length vectors, which will be fed into supervised prediction model.

#### Deep Feature Extraction

**Fig. 1:**
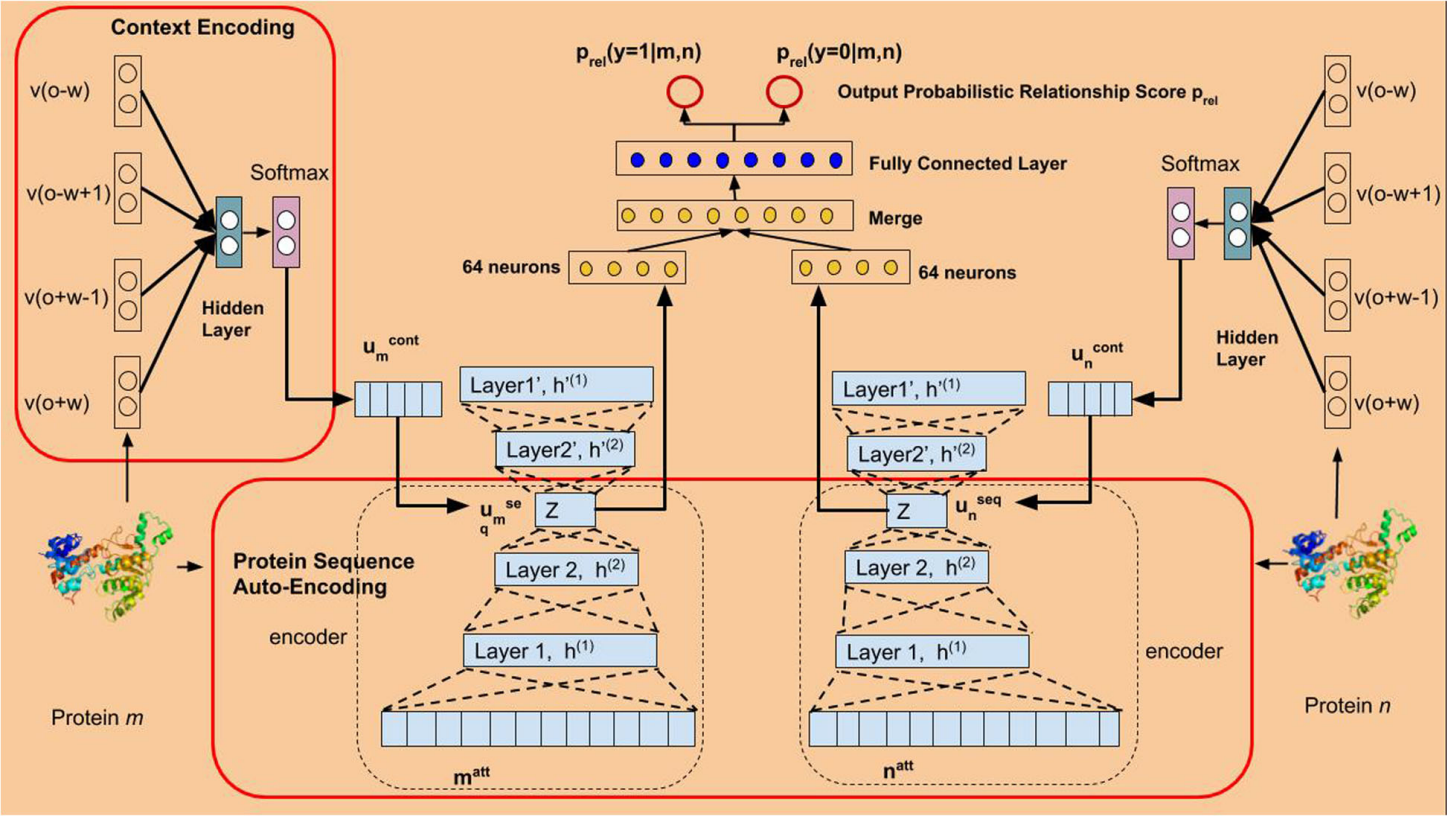
Multi-modal Deep Representation Learning Framework

To obtain the deep features from the 468 dimensional vectors, we utilize the Stacked Auto Encoder(SAE) framework as shown in Fig. 1 and in Eq. 6.

6 $$  \textbf{Map}_{seq}(\mathbf{p}_{i}) = \mathbf{v}_{i}  $$

First, the input layer in the SAE takes the protein *p*_*i*_’s feature vector **v**_*i*_ as the input vector generated during the data preprocessing phase. After *e* intermediate encoding layers, we obtain the output vector **h**^*out*^ as shown in Eq. 7 from the output layer. Here, $\mathbf {W}_{enc}^{k}\in \mathbb {R}^{n \times d_{k}}$ and $\mathbf {bias}_{enc} \in \mathbb {R}^{d_{k}}$ are the weight matrix and bias vector for the *kth* hidden layer in the encoding layers and *δ* represents the output activation function. *e* denotes the number of encoding layers. The output deep representation vector $\mathbf {h}^{out}\in \mathbb {R}^{d_{out}}$ of the input vector **v**_*i*_ is then projected back to the space of **v**_*i*_ using the decoding function through decoding layers. $\mathbf {W}^{k}_{dec}$ is the weight matrix for the *kth* decoding layer and *d* represents the number of decoding hidden layers. In our model, we choose the number of encoding layers the same as the number of decoding layers, i.e. *e*=*d*. Also, the number of hidden units in encoding layers equals to the number of hidden units in the decoding layers. During training phase, we update the parameters by using Stochastic Gradient Descent (SGD) methods to minimize the *L*_2_ loss function defined in Eq. 8. The whole deep feature extraction process can be depicted in Eq. 6, 7 and Algorithm 1.

7 $$ \begin{aligned} \mathbf{h}^{out} &= \delta\left(\mathbf{W}_{enc}^{k}\mathbf{v}_{i}+\mathbf{bias}_{enc}\right),k =1,2,3\dots e \\ \hat{\mathbf{v}}_{i} &= \delta\left(\mathbf{W}_{dec}^{k}\mathbf{h}^{out}+\mathbf{bias}_{dec}\right),k =1,2,3\dots d \end{aligned}   $$

8 $$  L_{2}(\mathbf{v}_{i},\hat{\mathbf{v}_{i}}) = (\mathbf{v}_{i}-\hat{\mathbf{v}_{i}})^{2} = (\mathbf{v}_{i}-(\mathbf{W}_{dec}\mathbf{h}+\mathbf{bias}_{dec}))^{2}  $$

#### CBOW Model Based On Metapaths

**Fig. 2:**
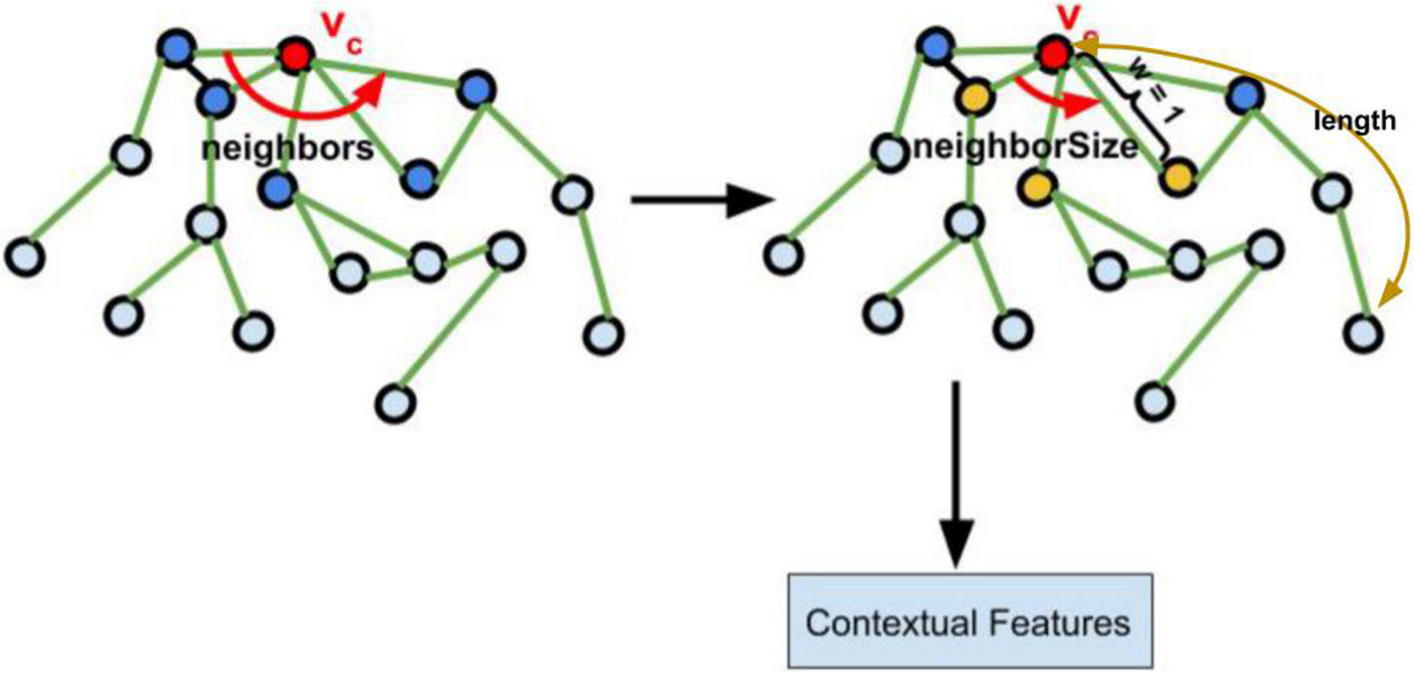
Metapath Generation


The CBOW(Continuous Bag of Words) model from natural language processing(NLP) techniques approximates the conditional probability in Eq. 9 [17]. Solving the optimization problem from Eq. 9 learns the distributive vectors that capture the proximity in the local network topology between nodes within the path length *w* as shown in Fig. 2. The objective function we try to minimize can be described in Eq. 9. In this paper, for a center node *e*_*c*_, we set *w*=1. Particularly, we only use the adjacent neighbors of node *e*_*c*_ as contextual nodes to maximize the structural context-local proximity. Here, the total number of protein nodes in the network is represented by *N* in Eq. 9. In the protein network, given a path consisting of protein nodes, *e*_1_→*e*_2_→*e*_3_→⋯→*e*_*l*_, we adopt CBOW model to minimize the negative log-likelihood function in Eq. 9. In our method, we define the CBOW model as the unsupervised model since we did not label the nodes manually. Instead, the system will automatically learn the representation vectors of each node.

9 $$  \begin{aligned} L(\theta) = -\frac{1}{N}\sum_{n=1}^{N} \sum_{\substack{-w \leq o\leq w \\ w \neq 0}} p(\mathbf e_{c} | \mathbf e_{c+o}; \theta) \end{aligned}  $$

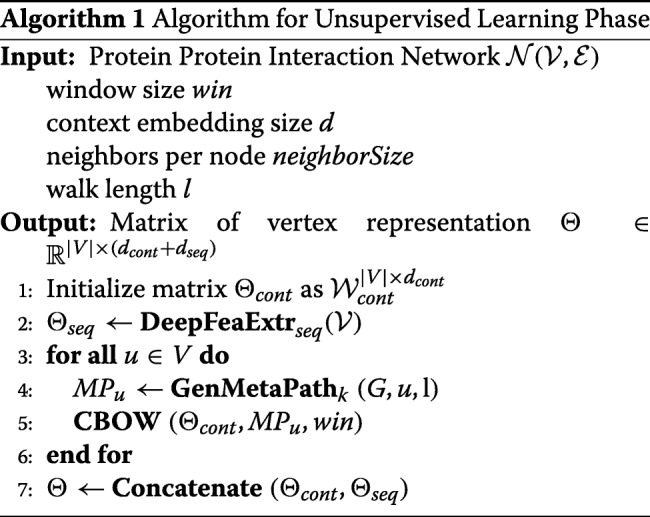



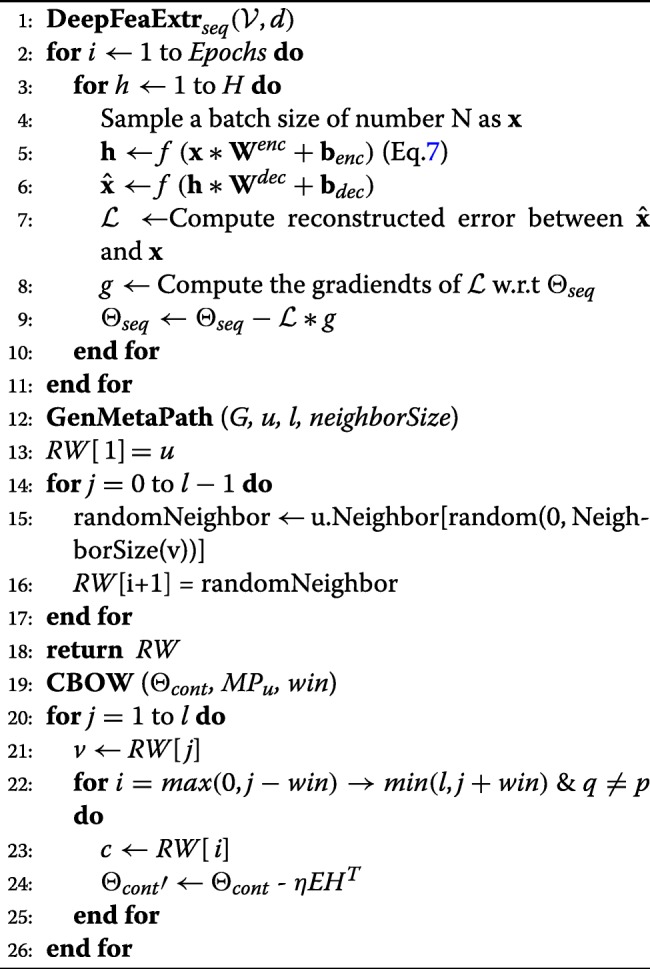


#### Homogeneous Metapaths Generation

Recently, thanks to the scalability and adaptability of random walk technique, lots of research methods utilize random walk based methods to learn node representations over graph structured data [17–19]. Among these methods, metapath is the most recent one. During metapaths generation process, we set the length parameter as *length* to indicate the walking distance starting from each protein node in the network. Also, for each protein node, we set the *neighborSize* as the contextual sampling parameters indicating how many neighbors we take into account as shown in Fig. 2. After that, we apply CBOW model trying to learn the distributed node representations within the network structured data and maximizes the likelihood of preserving the topological similarities between nodes. We can regard metapath-based methods as a graph representation model that estimates the occurrence likelihood of observing *v*_*i*_ given all the preceding vertices along the short path as shown in Eq. 10.

10 $$  Pr(v_{j} | v_{i-w},\dots,v_{i+w})  $$

The metapath approach presumes that within a network, the nodes co-occur along a short path tend to have intrinsic relationships. Therefore, based on random walk statistics [20, 21], metapath-based methods optimize the node embeddings such that nodes have similar representations if they co-occur on short random walks over the graph [22]. The basic idea of this set of approaches is to learn the encoding matrix such that the following equation is satisfied.

11 $$ \textbf{ENC}_{p}(\mathbf{z_{i},\mathbf{z}_{j}}) = \frac{e^{z_{i}^{T}z_{j}}}{\sum_{v_{k}\in \mathcal{V}}e^{z_{i}^{T}z_{k}}} \approx p_{\mathcal{G},\mathcal{T}}(v_{j}|v_{i})  $$

Here, *v*_*j*_ is the next neighboring node of *v*_*i*_. **ENC**_*p*_(*z*_*i*_**,z**_*j*_) represents the statistical probability of *v*_*j*_ given its neighboring node *v*_*i*_ along the path *p*. Since we only have one type of relationship or edge in the PPI networks, our metapaths generation process was defined as the homogeneous metapaths generation.

In our paper, the PPI networks are undirected graphs with vertices $\mathcal {V}$ representing proteins and edges $\mathcal {E}$ representing interactions. Accordingly, we generate node-oriented metapaths for the protein nodes in the PPI network first and then apply CBOW model to learn the distributed topological representations for each protein node. The details for the unsupervised learning phase can be found in Algorithm 1.

### Supervised Learning Phase

After the unsupervised learning phase, deep protein features and protein topological representations are learned. We then employ the feature fusion for those extracted features before feeding them as the inputs to the supervised learning model.

#### Feature Fusion

**Fig. 3:**
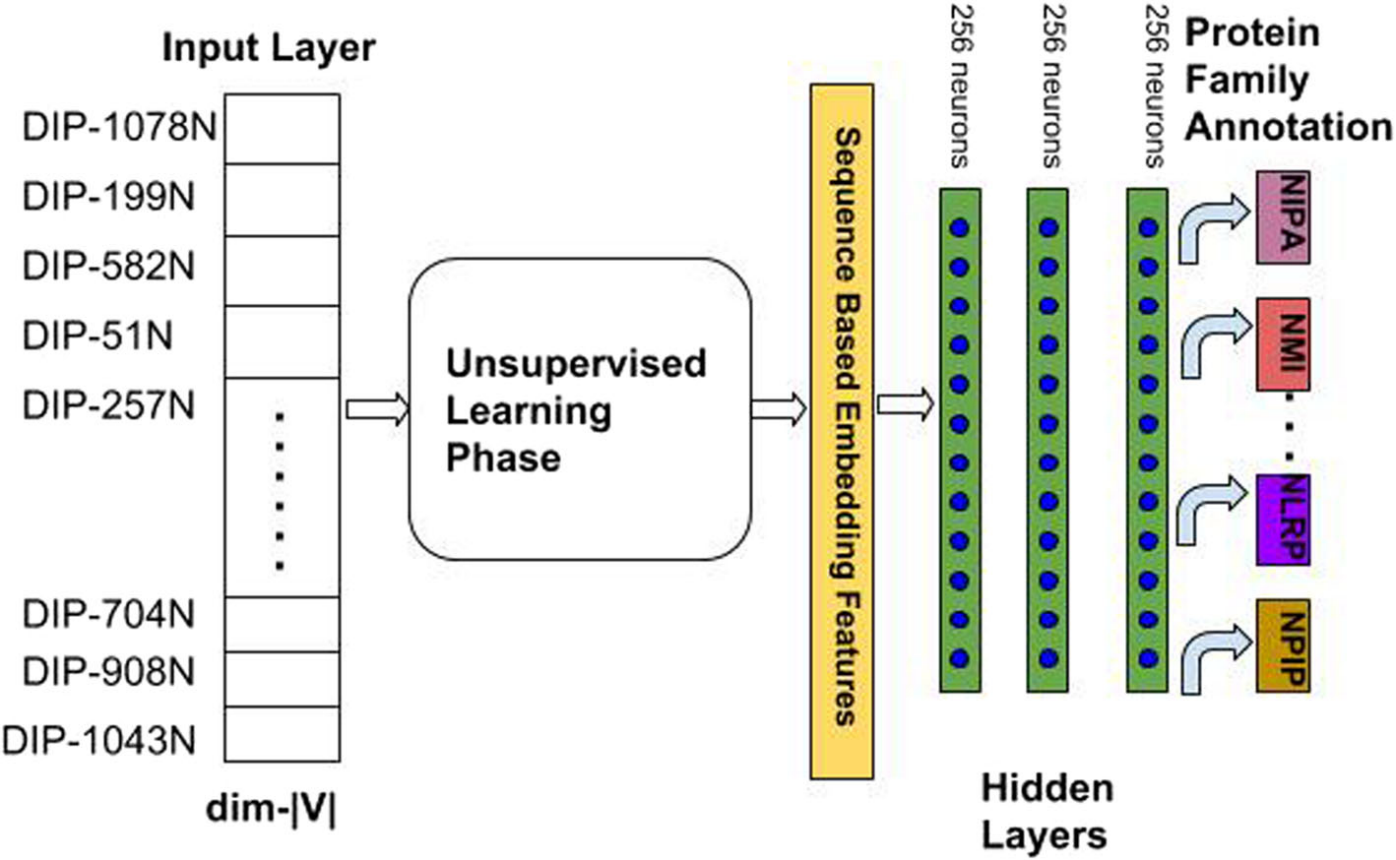
Protein Family Classification Deep Neural Network

Figures 1 and 3 present the integrated structure of our deep multi-modal representation learning framework, including the two phases of the leaning process. First, we fusion features learned from the CBOW model and SAE model. By doing this, various modalities as the outputs from the previous unsupervised learning phase are integrated.

12 $$\begin{array}{*{20}l}  \mathbf{h}_{u}^{out} &= \delta \left(\mathbf{W} \begin{bmatrix} u_{p}^{seq} \\ u_{p}^{cont} \\ \end{bmatrix} + \mathbf{b}\right) \end{array} $$

As shown in Eq. 12, given a protein *p*, its topological feature is $u_{p}^{cont}$ and its deep protein sequence feature is $u_{p}^{seq}$ respectively. We concatenate these two features together as *u*_*p*_ to represent *p*.

#### Supervised Learning Model

After we concatenate both topological proximity representations and deep physicochemical features of the proteins, we use them for the downstream protein interaction identification and protein multi-family classification tasks. Given two proteins *m* and *n*, their deep sequence features are represented by vectors $u_{m}^{seq}$ and $u_{n}^{seq}$ respectively. Their topological proximity features are represented as $u_{m}^{cont}$ and $u_{n}^{cont}$, which are obtained by *CBOW* model based on generated metapaths. For the CBOW model, we set the window size *win*=1 and the walking length as *l*. After this unsupervised learning process, we concatenate the obtained features as *u*_*m*_ and *u*_*n*_ and feed them into the supervised learning model. Then, the supervised learning model uses these features to perform PPI identification and Protein Multi-Family classification tasks as shown in Figs. 1 and 3.

- The PPI identification model consists of two deep neural networks separately as shown in Fig. 1. One is for protein *m*, and the other is for protein *n*. After the last layer, we combine the extracted lower dimensional features of the two proteins and feed those features into the fully connected layer connected to the output layer for classification. During the learning process, we use the binary cross entropy Eq. 13 as the loss function since the interaction can only exist or not. Therefore, the final classification results are the probability $\hat {y} = p(y|m,n)$ that the two given proteins *m* and *n* interact with each other.
  13 $$  {}\mathcal{L}(\hat{y},y) = -\frac{1}{N}\sum_{i=1}^{N}[y_{i}log\hat{y_{i}}+(1-y_{i})log(1-\hat{y_{i}})]  $$
- For classifying the protein families, we construct the model as shown in Fig. 3 and utilize the same features obtained from the unsupervised learning phase. We employ deep neural networks(DNN) for extracting non-linear hidden features. Since we are required to classify proteins into multiple categories, we use the categorical cross entropy Eq. 14 as the loss function here. Since this is multi-class classification task, we calculate the individual loss rate for every class label *c* per observation *o* and sum the results over all of the *N* training samples.
  14 $$  \mathcal{L} = -\frac{1}{N}\sum_{i=1}^{N}\sum_{c=1}^{C} y_{o,c}[log(\hat{y}_{o,c})]  $$

During the experiments, we use stochastic gradient descent(SGD) as the optimization method.

## Results

In the unsupervised learning phase, we set the the hidden layer parameters for the stacked auto-encoder(SAE) as 256−128−64−128−256. 256,128,64,128 and 256 represent the number of neurons for each hidden layer separately. During the SAE training phase, we use the mean squared error(MSE) as the loss function defined in Eq. 15. After the auto-encoding process, the protein sequence vectors are projected to the lower dimensional space with vector length 64 at the layer ’z’ in Fig. 1. For the graph topology embedding, during the metapath generation process, we fix the contextual sampling parameter *neighborSize*=4 and the metapath length *l*=10 to generate the metapaths starting from each protein node. Then, we set the window size *win*=1 and the node vector length *v*=128 for each protein such that the CBOW model is able to learn the distributive node representations.

15 $$  MSE = \frac{1}{n}\sum_{i=1}^{n}(Y_{i}-\hat{Y_{i}})^{2}  $$

### Dataset Description

During the experiments, we used two complete datasets including Database of Interacting Proteins (DIP) released 20170205_FULL dataset http://dip.mbi.ucla.edu/dip/ and Human Protein Reference Database http://www.hprd.org/ (HPRD), which are the benchmarks and complete databases most methods were tested on. The DIP dataset includes eight species *D. melanogaster, S. cerevisiae, E. coli, C. elegans, H. sapiens, H. pylori, M. musculus, R. norvegicus*. After removing the duplicate protein sequences and the self-interactions, we obtained 3790 PPIs for C.elegans, 22,067 for D. melanogaster, 11,521 for E.coli, 1358 for H.pylori, 6677 for H.apiens, 2385 for M.musculus, 523 for R.norgegicus and 22,502 for S.cerevisiae. First, we convert the raw protein data with various sequence lengths into 486 equal length vectors using the computational methods defined in the “Methods” section. Then, we generated the negative datasets from eight different subcellular locations including *Cytoplasm, Nucleus, Endoplasmic reticulum, Golgi apparatus, lysosome, Mitochondrion, Cell Membrane and Lipid-ancho*[10], in which different species of proteins reside. After that, we generated the corresponding negative samples by randomly matching those proteins with others found in the different subcellular locations. To avoid biased data, we generate the equal number of negative samples as the positive samples. The subcellular location information can be accessed from the UniProt database https://www.uniprot.org/locations/. After constructing the data, we mixed and shuffled the data for each species in the DIP dataset. Then, we split the data into the training dataset and testing dataset with the ratio of 80% and 20% respectively.

**Fig. 4:**
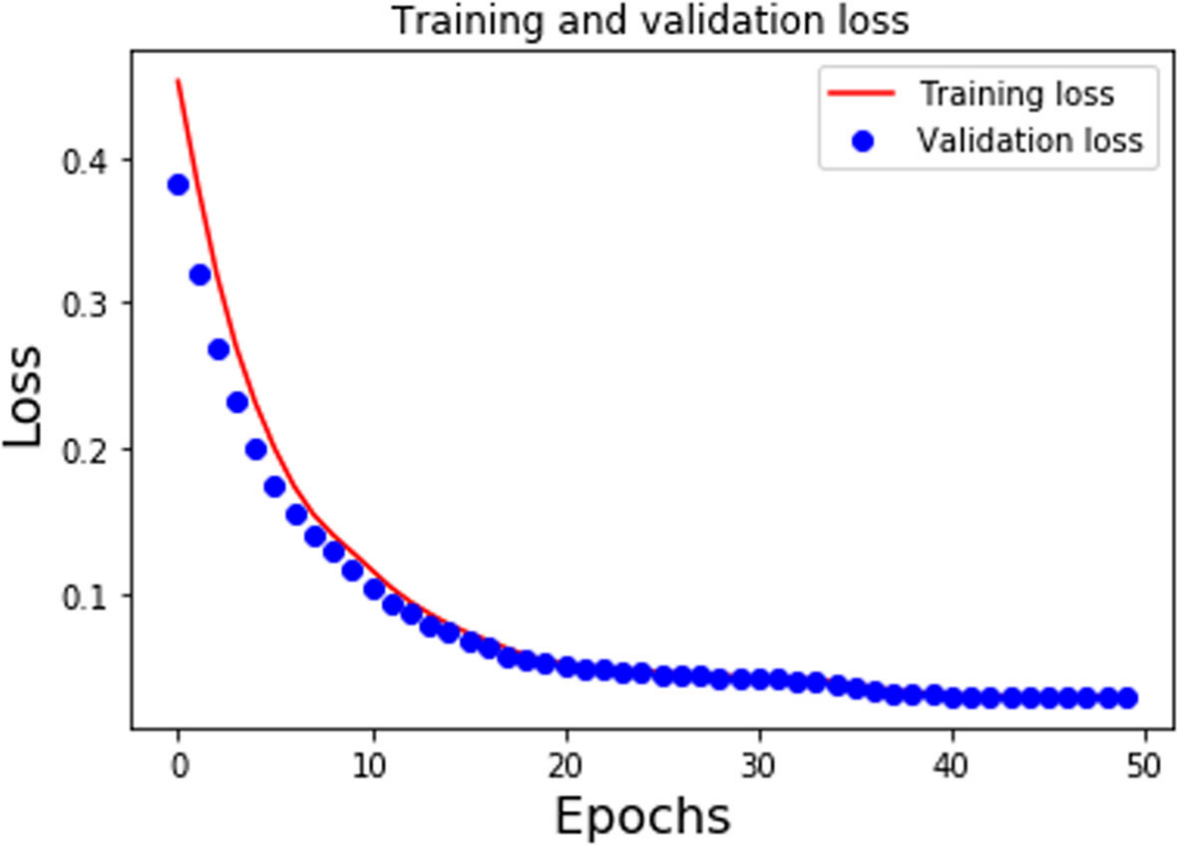
SAE Loss for S.cerevisiae Species

During the training phase, for each species, we used the PPIs in the training dataset to generate metapaths. The PPIs in the testing dataset are hold-out. We trained the CBOW model for 10 epochs and the SAE model for 50 epochs during the unsupervised learning phase. Figure 4 presents the MSE loss of training dataset and validation dataset for *S.cerevisiae* species during the training process using SAE framework. From the result, it can be seen that the validation loss and the training loss are synchronized with each other. This indicates that our model is not overfitting: the validation loss is decreasing instead of increasing, and there is rarely any gap between the training and validation loss. For the CBOW model, we give an example in Table 1 after we train with the *H.sapiens* dataset. The float values indicate the cosine similarity between the query protein node and the top-10 most similar protein nodes in descending order. Given a query protein with ID DIP-41844N, which is the protein *5-hydroxytryptamine receptor 2A*, we returned the most similar proteins measured by cosine similarity with respect to the query. The returning results can be verified by checking actual neighbors of DIP-41844N in the DIP database. It turns out that all the 1-hop neighbors of DIP-41844N have been correctly returned by the CBOW model ranked by their similarity scores. After the unsupervised learning phase, we performed late fusion on these deep abstract sequence vectors and topological feature vectors as 128+64=192 length vectors. Then, we feed them into the supervised learning model for downstream interaction identification and multi-family classification tasks. For the interaction identification task, the supervised learning model consists of one fully connected layer having 64 hidden units. The number of units in the output layer is decided by the number of classes we need to identify. For the protein interaction identification task, the class label is either 0 or 1 indicating interaction or non-interaction respectively. For the family classification task after the unsupervised learning phase, we build three-layer deep neural network(DNN) as shown in Fig. 3. We train the DNN for 200 epochs with a dropout rate at 0.5 and batch size at 64. The number of units in the output layer is determined by the number of protein families we aim to categorize.

**Table 1:** Top-10 Similar Proteins to DIP-41844N(5-hydroxytryptamine receptor 2A)

Protein ID	Protein Name	Cosine Similarity
DIP-49960N	Nucleoside diphosphate kinase 3	0.90755
DIP-31554N	Ribosomal protein S6 kinase alpha-3	0.89557
DIP-38298N	NADH dehydrogenase [ubiquinone] 1 beta subcomplex subunit 10	0.85293
DIP-36377N	Microtubule-associated protein 1A	0.82932
DIP-61575N	Cannabinoid receptor 1	0.82696
DIP-41406N	Ankyrin repeat and sterile alpha motif domain-containing protein 1B	0.82623
DIP-61135N	39S ribosomal protein L28, mitochondrial	0.82200
DIP-5723N	neurotrophin-3 receptor precursor	0.81994
DIP-61136N	Serum paraoxonase/arylesterase 2	0.81177
DIP-59826N	Metabotropic glutamate receptor 2	0.79176

### Evaluation Metrics

During the experiments, we used Area Under the Receiver Operating Characteristic curve(AUC_ROC), Specificity(SPC), Accuracy(ACC), Precision, and Recall (or Sensitivity) to measure the prediction accuracy and data divergence using our method. The metric formulas are described as the following equations:

16 $$\begin{array}{@{}rcl@{}} Precision &=& \frac{TP}{TP+FP} \end{array} $$

17 $$\begin{array}{@{}rcl@{}} ACC &=& \frac{TP+TN}{TP+TN+FP+FN} \end{array} $$

18 $$\begin{array}{@{}rcl@{}} SPC &=& \frac{TN}{TN+FP} \end{array} $$

19 $$\begin{array}{@{}rcl@{}} Recall &=& \frac{TP}{TP+FN} \end{array} $$

Here, TP, FP, TN, and FN denote True Positive, False Positive, True Negative and False Negative respectively.

### Comparison with traditional methods

**Fig. 5:**
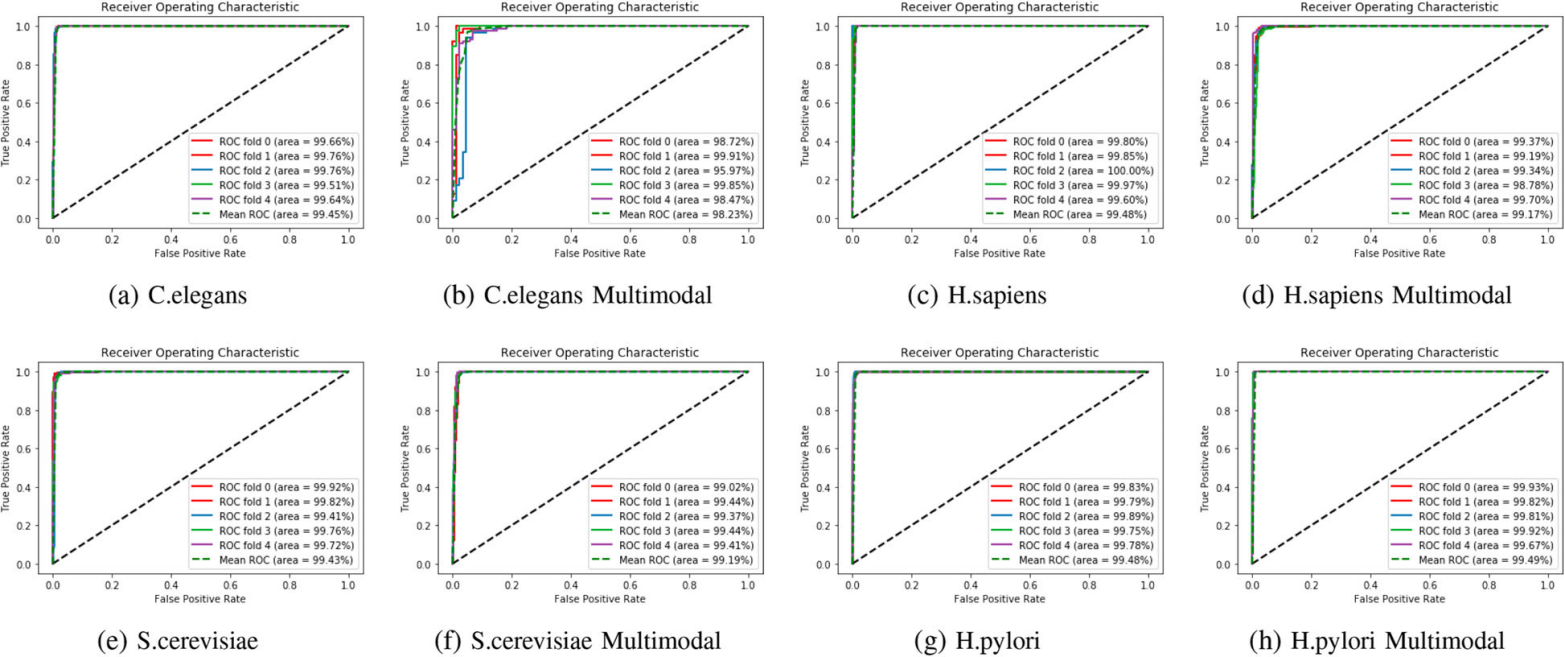
5-CV AUC-ROC score for eight species

**Fig. 6:**
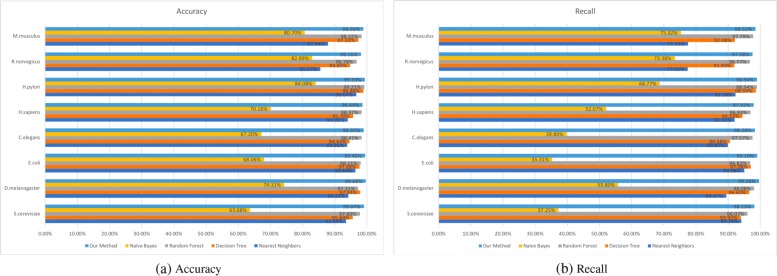
**a** Comparison of ACC score between our method and traditional machine learning techniques over eight species **b** Comparison of Recall score between our method and traditional machine learning techniques over eight species

**Fig. 7:**
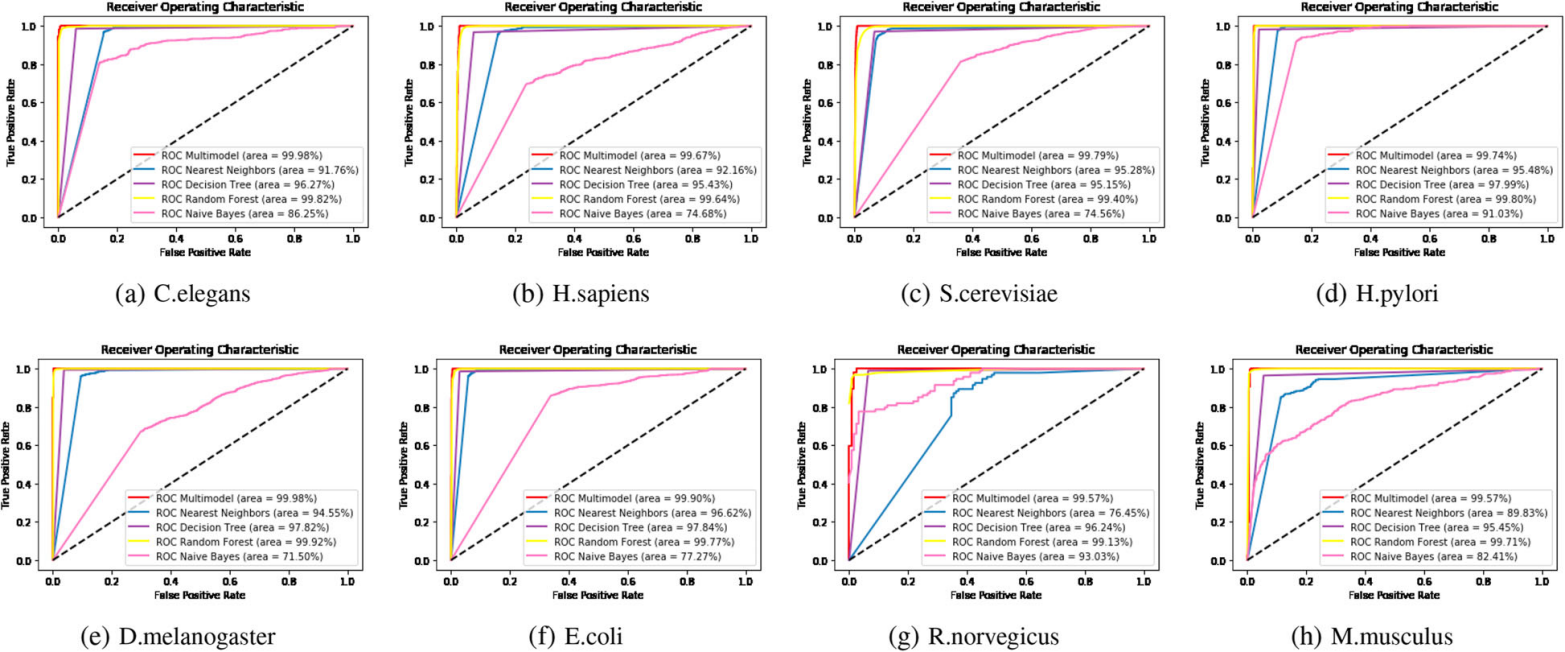
Comparison of the AUC-ROC score between our method and traditional machine learning methods over eight species

In our paper, we extensively compare our multi-modal deep representation learning framework with the traditional machine learning methods. We present the results of our method using all eight species in the DIP dataset and assess the receiver operating characteristics(ROC) scores using 5-Cross Validation methods in Fig. 5. Since the number of neurons in each hidden layer, the number of layers, and the vector size of the metapath representations of proteins are all critical parameters, we studied and tried various combinations to discover the model with the best performance. After that, the model with the best performance was selected to test the hold-out dataset as shown in Table 2. From the results in Fig. 5, we can see that most of the AUC scores achieved 0.99 using our model. To evaluate the performance of our method more thoroughly, we compared our model with traditional machine learning techniques[23, 24] including Nearest Neighbors(k=2), Decision Tree, Random Forest and Naive Bayes in Figs. 6 and 7 respectively using ACC, Recall and AUC-ROC metrics.

**Table 2:** Hyper-parameter settings

Parameter	Settings
Batch Size	64
Learning Rate	0.01
Stacked Auto Encoder Architecture	256-128-64-128-256
Optimization Method	SGD
Window size	1
CBOW Node Embedding Size	128
Neighboring Node	4
Metapath Length	10

### Comparison with state of the art methods

We also compared our model over the DIP dataset across different species with the cutting-edge methods comprising of deep learning techniques using different evaluation metrics. Since previous researches use different species for evaluation, we compare them separately as shown in Tables 3, 4, 5, 6, and 7. For instance, for S.cerevisiae species, compared to the other four most advanced methods, our multi-modal deep learning predictor still outperforms them. The ACC, Precision, Recall and AUC scores reach 2.76*%*, 4.27*%*,5.73*%* and 0.0158 higher than Du’s work, which proves the advantage of our model. And for E.coli, Drosophil and C.elegans datasets, we compare our model with two other most advanced methods [10] and [25] using the same metrics including Recall, ACC and SPC. For E.coli and C.elegans species, we outperform them using all three metrics. While, for Drosophil species we achieve higher performance using ACC and SPC metrics but slightly lower than other two methods using the Recall metric.

**Table 3:** Comparison of 5-CV prediction performance between our method and state of the art methods using S.cerevisiae dataset(Note: N/A: Not Available)

Method	Precision	ACC	Recall	AUC
Our Method	100.00% ±0.00%	99.08% ±0.13%	98.15% ±0.27%	0.9908 ±0.13
Du’s work[9]	96.65% ±0.59%	94.43% ±0.30%	92.06% ±0.36%	0.9754
Wong’s work[26]	96.45% ±0.45%	91.10% ±0.31%	93.92% ±0.36%	0.94 ±0.002
You’s work[27]	91.94% ±0.62%	91.36% ±0.36%	90.76% ±0.69%	0.9707 ±0.12
Guo’s work[3]	88.87% ±6.61%	89.33% ±2.67%	87.37% ±0.22%	N/A

**Table 4:** Training performance between our method and other methods over the E.coli species(5-CV)

Method	Recall	ACC	SPC
Our Method	97.20%	97.85%	99.08%
Sun’s work[10]	96.89%	96.05%	95.28%
Guo’s work[25]	95.11%	92.73%	90.35%

**Table 5:** Training performance between our method and other methods over the Drosophil species(5-CV)

Method	Recall	ACC	SPC
Our Method	99.06%	99.20%	99.78%
Sun’s work[10]	99.51%	97.84%	96.28%
Guo’s work[25]	99.53%	90.09%	80.65%

**Table 6:** Training performance between our method and other methods over the C.elegans species(5-CV)

Method	Recall	ACC	SPC
Our Method	98.28%	98.81%	100.00%
Sun’s work[10]	99.35%	97.23%	95.28%
Guo’s work[25]	96.46%	97.51%	98.55%

**Table 7:** Prediction accuracy comparison between our method and state of the art methods over the H.sapiens and HPRD dataset

Method	HPRD	DIP
Our Method	97.61%	95.94%
Sun’s work[10]	97.14%	93.77%
Pan’s work[30]	86.70%	90.04%

### Prediction Across Species

We not only tested our model within the same species, but also used the *S.cerevisiae* training dataset as the overall training dataset and assess the prediction performance on the rest seven species using various metrics. In the experiment, the *S.cerevisiae* training dataset includes 36,006 negative and positive samples. The prediction performance of the rest seven species is presented in Table 8. We can see from the table that the accuracy for *D. melanogaster, E. coli, C. elegans, H. sapiens, H. pylori, M. musculus, R. norvegicus* are 96.76*%*,97.70*%*,98.44*%*,98.50*%*,98.84*%*,98.69*%*,99.77*%* respectively. Consequently, although only using single species training dataset, our multi-modal deep representation learning framework is still outperforms other methods using various evaluation metrics. Furthermore, we also examines D. melanogaster species and R. norvegicus species, which have not been explored by other methods yet and also achieves promising prediction accuracy.

**Table 8:** Prediction Results on Seven Species using Our Proposed Framework, Based on S.cerevisiae Training Dataset as the Overall Training Dataset (Note: N/A: Not Available)

Species	Our Method	Du’s work[9]	Huang’s work[28]	Zhou’s work[2]
*C.elegans*	98.44%	94.84%	81.19%	75.73%
*H.sapiens*	98.50%	93.77%	82.22%	76.27%
*M.musculus*	98.69%	91.37%	79.87%	76.88%
*H.pylori*	98.84%	93.66%	82.18%	N/A
*D.melanogaster*	96.76%	N/A	N/A	N/A
*E.coli*	97.70%	92.19%	66.08%	71.24%
*R.norvegicus*	99.77%	N/A	N/A	N/A

### Prediction using HPRD dataset

**Fig. 8:**
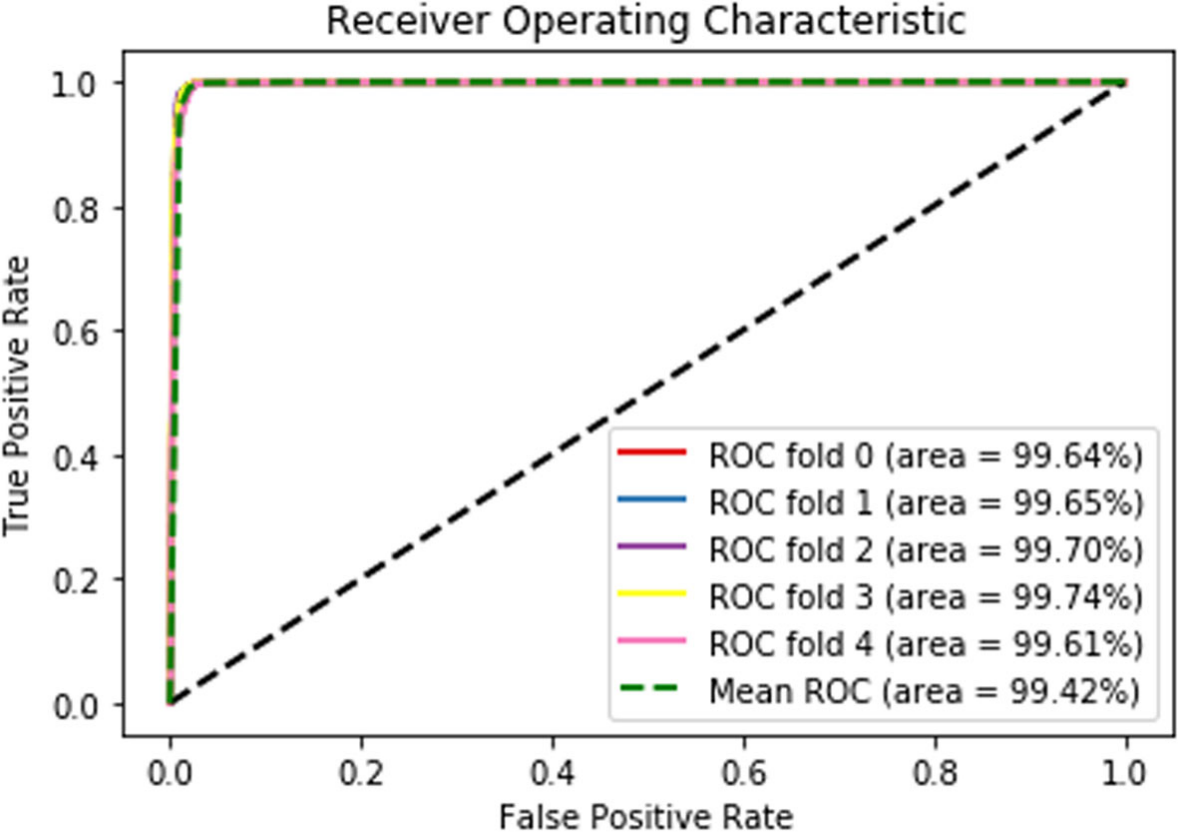
5-CV AUC-ROC score for HPRD dataset

To compare other methods comprehensively and extending our previous work[29], we used HPRD as another benchmark dataset for testing. For the HPRD dataset, we only retrieved human proteins with family information from the Uni-Prot database while disgarded the human proteins without family annotations. After that, we have 16,915 human PPI interactions and 4185 human proteins. Then, we performed PPI prediction on the HPRD dataset. During this process, we generated the same amount of negative instances with positive samples using five subcellular positions consisting of *Cyptoplasm, Endoplasimic reticulum, Golgi apparatus, Lysosome, Mitochondrion, Nucleus*. The five cross validation ROC curve is plotted in Fig. 8. We compared our method with [10] and [30] using the same DIP H.sapiens dataset in Table 7 and the HPRD dataset and proved that our prediction accuracy is higher than those methods.

### Protein Family Classification

**Fig. 9:**
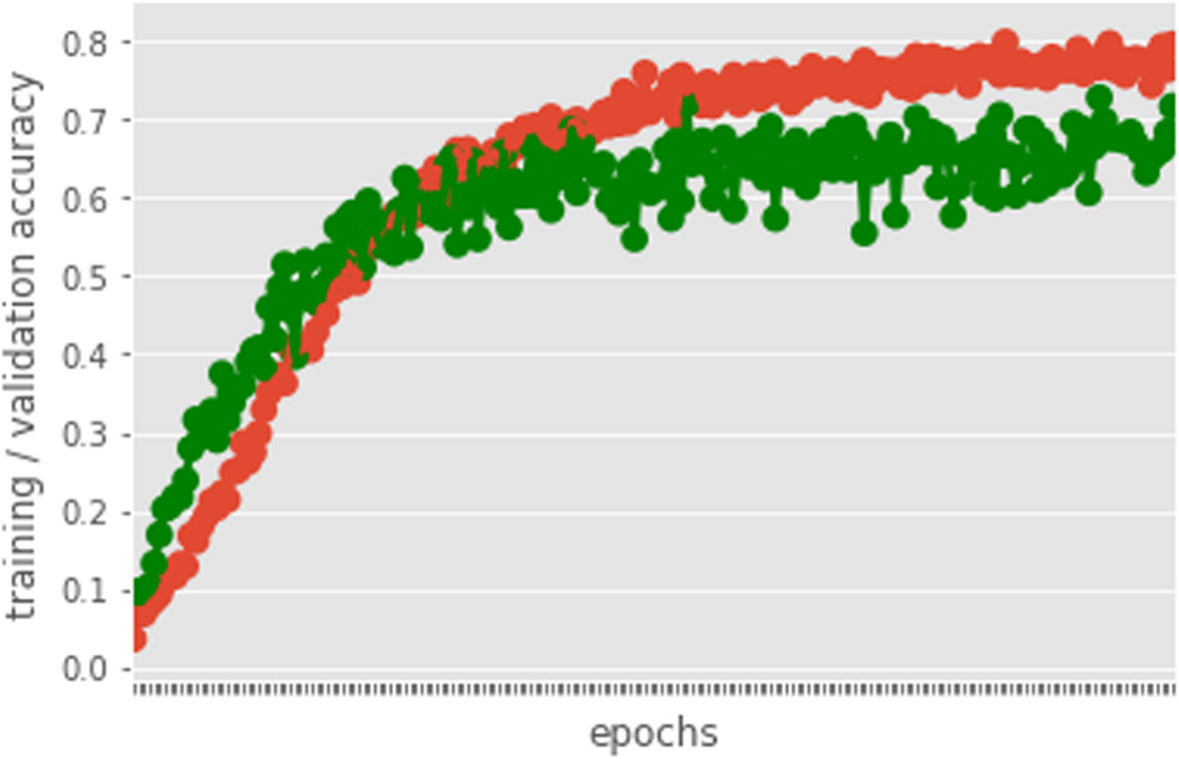
Training and Validation Accuracy

In addition to interaction prediction, we performed downstream multi-family protein classification tasks as well using the same features from the unsupervised learning phase. In our experiments, family annotations are obtained from the UniProt database https://www.uniprot.org/. We use all the proteins in the DIP dataset and acquire the families they belong to from the database. Amongst the protein families in the dataset, we only choose those families with more than 15 samples. This results in the top frequent 99 protein families to verify our results. We present the training accuracy and validation accuracy in Fig. 9 to show our model is not subjected to overfitting. Then, we evaluate using 5-CV and compare our prediction accuracy with traditional methods including Random Forest, SVC and GaussianNB classifiers. Since classifying proteins according to their family annotations is a multi-class classification task, we therefore use F1 score to assess the models’ performance defined as the following Eq. 20:

20 $$  F1 = 2\times \frac{precision\times recall}{precision+recall}  $$

**Fig. 10:**
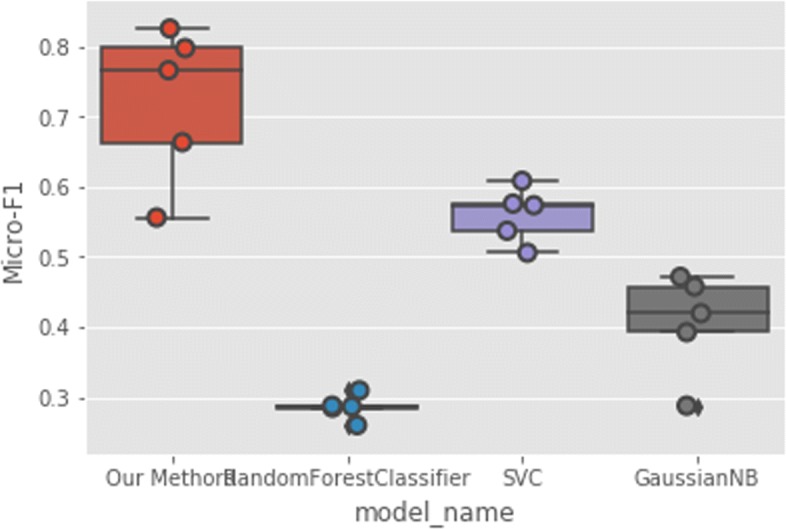
Protein Multi-Family Classification Micro-F1 Score

**Fig. 11:**
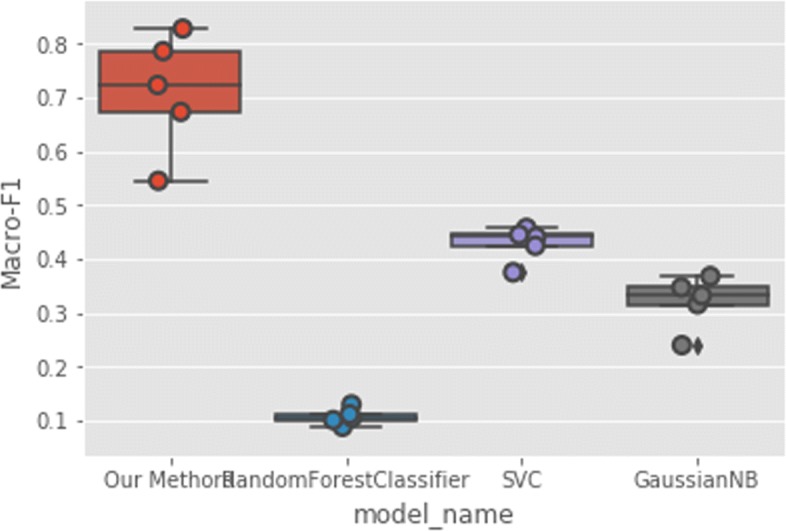
Protein Multi-Family Classification Macro-F1 Score

As can be seen in Figs. 10 and 11, our multi-modal deep representation learning framework outperforms the traditional methods using both Micro-F1 and Macro-F1 scores.

## Discussion

In this paper, we compare our multi-modal deep learning framework with representative traditional machine learning methods and state of the art methods. These state of the art methods include deep learning methods. Then, we verify our method across all eight species provided by DIP_FULL and HPRD datasets. For instance, for the *S*.*cerevisiae* dataset in DIP, our accuracy achieved 99.79*%* while the performance of the other four methods was 95.28*%*,95.15*%*,99.40*%* and 74.56*%* respectively. As for the Recall score for *S*.*cerevisiae* species, our recall scores achieved 98.13*%* while the values of the other four methods are 97.07*%*,93.92*%*,94.14*%* and 37.25*%* respectively. For the HPRD dataset, we can see from the results that the mean ROC score achieves 0.9942, which is consistent with other species in the DIP dataset.

Additionally, we predict protein families based on the deep protein representation features with our models on the DIP dataset. Our method achieves up to 33.9% improvements in terms of Micro-F1 score and achieves up to 74.4% improvements in terms of Macro-F1 score over the best performance among Random Forest, SVC and GaussianNB classifiers. Different from other protein family classification methods[31–33] which require at least 200 instances for each family, our method does not heavily rely on large dataset.

## Conclusions

In summary, our multi-modal deep representation learning framework harvest features that are highly predictive of protein function. It captures both sequential protein raw information with the topological structure to improve the PPI prediction accuracy and multi-class classification accuracy given the complex, non-linear interaction networks PPI network. We apply our methods on both DIP and HPRD datasets. After applying the CBOW model based on generated metapaths, our model is able to take into account the graph topological information into account. We use various mainstream metrics to assess the performance over the new released DIP_20170205 FULL dataset including eight species and HPRD datasets. Through extensive comparisons with both traditional machine learning methods and state of the art deep learning methods, we prove that our method outperforms most of them over the same datasets.

## Data Availability

The DIP dataset is publicly available at http://dip.mbi.ucla.edu/dip/. The HPRD dataset is publicly available at http://www.hprd.org/. The UniProt dataset is publicly available at https://www.uniprot.org/locations/. The source code is available upon request.

## Abbreviations

AUC: Area under curve CBOW: Continuous bag-of-words
DNN: Deep neural networks
DT: Decision trees
FN: False negative
FP: False positive
NB: Naive bayes
NN: Nearest neighbor
ROC: Receiver operating characteristics
SAE: Stacked Auto Encoder
SGD: Stochastic gradient descent
TN: True negative
TP: True positive
